# No impairment of monocyte-derived Langerhans cell phenotype or function in early-onset psoriasis

**DOI:** 10.1111/j.1365-2230.2011.04172.x

**Published:** 2012-01

**Authors:** F L Shaw, I Kimber, R Begum, M Cumberbatch, R J Dearman, C E M Griffiths

**Affiliations:** * Toxicology Group, Faculty of Life Sciences, University of ManchesterManchester, UK; † Dermatological Sciences, Salford Royal NHS Foundation Trust, Faculty of Medical and Human Sciences, University of Manchester, Manchester Academic Health Science CentreManchester, UK

## Abstract

**Background:**

Migration of epidermal Langerhans cells (LCs) in response to the cytokines interleukin (IL)-1β and tumour necrosis factor (TNF)-α is impaired in uninvolved skin of patients with early-onset psoriasis.

**Aim:**

To investigate whether this impairment is a reflection of a systemic defect in dendritic cells (DCs), using an established model of monocyte-derived LC-like cells (mLCs).

**Methods:**

CD14+ monocytes isolated from both patients with psoriasis and healthy control volunteers were cultured in a cytokine cocktail for 5 days to promote their differentiation into mLCs, then stimulated for 24 h with TNF-α, IL-1β (both 100 ng/mL) or medium alone. Cellular surface protein expression was quantified by flow cytometry, and the ability of cells to migrate to media supplemented with C-C motif ligand (CCL)19 was assessed using a Transwell migration assay. The cytokine and chemokine content of supernatants was analysed by cytokine array.

**Results:**

CD14+ cells acquired an LC-like phenotype with high expression of CD1a and major histocompatibility complex (MHC) class II. There were no differences in the expression of activation markers or in the secretion of cytokines by mLCs isolated from patients with psoriasis and those isolated from healthy controls. Moreover, mLCs isolated from both groups displayed comparable ability to migrate *in vitro*.

**Conclusions:**

These data suggest that the failure of LCs to migrate in response to stimulation in patients with psoriasis is not attributable to a systemic defect in DC function, but is rather a reflection of local changes in the epidermal microenvironment.

## Introduction

Psoriasis is a chronic, inflammatory skin disorder that affects approximately 2% of the population. It is considered to be an immune-mediated disease, and consistent with this is the observation that immunosuppressants modulating T-cell function serve as effective therapies for psoriasis.^1^–^4^ More recently, attention has focused on the role of dendritic cell (DC) subtypes in the pathogenesis of psoriasis. Some DC subtypes, such as plasmacytoid DCs, are found only in plaques,^5^–^7^ whereas other subsets are also present in the skin of healthy individuals, but are at an increased number in plaques, such as CD11c+ cells that express both inducible nitric oxide synthase and tumour necrosis factor (TNF)-α.^8^

Abnormalities in the function of DCs are not merely confined solely to plaques of psoriasis. We reported previously that the function of Langerhans cells (LCs) within uninvolved, clinically normal skin of patients with early-onset (presenting before the age of 40 years) psoriasis differs markedly from the LCs of healthy individuals. LCs are a population of skin-resident DCs that reside in the epidermis. One of their functions is to capture and process external antigens encountered at the skin surface, and respond to such challenges (in the presence of additional signals) by migrating via afferent lymphatics to the skin-draining lymph nodes, where they present antigen to T cells. The mobilization of LCs is induced primarily by the proinflammatory cytokines TNF-α and interleukin (IL)-1β. It is believed that following stimulation, LCs produce IL-1β, and that this, in addition to providing one signal for migration, also induces adjacent keratinocytes to produce TNF-α, which provides a second independent signal for migration.^9^ Exposure via intradermal injection to either of these cytokines, or topical exposure to contact allergens, induces LC migration from the epidermis in healthy individuals. However, in the uninvolved skin of patients with early-onset psoriasis, the ability of LCs to migrate in response to topical exposure to the contact allergen diphenylcyclopropenone, and to the cytokines TNF-α and IL-1β, is absent.^10^

It has been shown in mice that under steady-state conditions, epidermal LCs are repopulated by resident proliferating precursors,^11^ a process that has recently been suggested to be dependent on transforming growth factor (TGF)-β.^12^ However, under inflammatory conditions, such as UV exposure, bone marrow-derived DCs have been demonstrated to act as precursor cells.^11^ Little is known about the repopulation of LCs in human skin, but Geissmann *et al*. first reported in 1988 that peripheral blood CD14+ monocytes differentiate into LC-like cells (monocyte-derived LC-like cells; mLCs) following short-term culture in the presence of TGF-β, IL-4 and granulocyte–macrophage colony-stimulating factor (GM-CSF).^13^ Migratory CD14+ DCs are also able to differentiate into mLCs in the presence of TGF-β.^14^ Ginhoux *et al*. expanded upon this work, demonstrating that LCs arise from monocytes *in vivo*, and that monocytes with high expression of granulocyte differentiation antigen 1 (Gr-1^hi^ monocytes) are the immediate circulating precursors of LCs.^15^ It is legitimate therefore to consider whether the abnormal function of LCs in the uninvolved skin of patients with early-onset psoriasis is associated with systemic changes reflected by the behaviour of mLCs.

In the present study, we compared and contrasted the phenotype and function of mLCs from healthy subjects and from patients with early-onset psoriasis.

## Methods

The study was approved by Salford and Trafford Research Ethics Committee (08/H1004/61), and written informed consent was obtained from all participants.

### Subjects

This was a laboratory investigation comparing a patient group with a control group matched for age and gender.

Patients of either gender aged 18–55 years of age with no current febrile illness were enrolled in the study.

Exclusion criteria were: presence of atopy or allergy, and use of regular systemic medication within the 4 weeks prior to study enrolment.

Patients on topical therapies were not excluded; however, our patients presented with mild to moderate psoriasis, and the application of active topicals apart from emollients, if used at all, was minimal.

In total, 12 patients with early-onset psoriasis (8 women, 4 men; mean ± SD age 37.9 ± 2.8, range 21–52) and 12 healthy controls (7 women, 5 men; age 36.2 ± 2.8; range 19–46) were enrolled in the study.

The clinical severity of patients with early-onset psoriasis was measured using the Psoriasis Area and Severity Index (PASI^16^), and ranged from 1.3 to 29.1.

Blood samples (120 mL) were taken from each participant, drawn into 10-mL vacuum test-tubes coated with 171 IU lithium/heparin (Southern Syringe, Manchester, UK), and used for cell isolation.

### Monocyte isolation

CD14+ cells were isolated from whole blood using a magnetic-activated cell sorting (MACS) negative selection system according to the manufacturer's instructions (Miltenyi Biotec, Bisley, UK).

### Dendritic cell culture

CD14+ cells were cultured in flat-bottomed 24-well plates at a density of 1 × 10^6^ cells/mL for 5 days in RPMI 1640 culture medium containing 10% heat-inactivated fetal calf serum (FCS), 100 U/mL streptomycin, 100 μg/mL penicillin and 292 μg/mL L-glutamine (complete RPMI; Invitrogen, Paisley, Renfrewshire, UK). The medium was supplemented with 10 ng/mL TGF-β, 250 ng/mL GM-CSF and 10 ng/mL IL-4 (R&D Systems, Abingdon, Oxfordshire, UK). Cells were fed on day 3. On day 5, cells were resuspended in complete RPMI supplemented with 250 ng/mL GM-CSF and 100 ng/mL IL-4 at a density of 1 × 10^6^ cells/mL, and stimulated with medium alone (vehicle control), TNF-α or IL-1β (both 100 ng/mL; R&D Systems, Abingdon, UK) for 24 h. The cells were not cultured with TGF-β at this stage so as to enable LC maturation in response to the inflammatory mediators.^17^ Following stimulation, cells were removed from the plate by gentle pipetting, and centifuged at 200 ***g*** for 5 min. The supernatants were aspirated and stored at −80 °C for measurement of cytokines and chemokines. The resulting cells were resuspended either in 5% FCS in phosphate-buffered saline for flow cytometric analysis, or in complete RPMI at 2 × 10^6^ cells/mL for use in the migration assay.

### Flow cytometry

Cells were analysed for expression of CD14, CD54, CD86, major histocompatibility complex (MHC) class II and CD1a via indirect staining (FACSCalibur; Becton Dickinson, Oxford, Oxfordshire, UK). Dead cells were excluded by staining with 5 μg/mL propidium iodide (Sigma-Aldrich, Poole, Dorset UK) immediately before analysis. Data were analysed using FlowJo software (Tree Star Inc., Ashland, MA, USA).

### Luminex analyses

Concentrations of cytokines and chemokines [IL-10, interferon-γ-induced protein (IP)-10, macrophage inflammatory protein (MIP)-1α and TNF-α] within culture supernatants were measured by cytokine array (BioPlex, Bio-Rad, Hemel Hempstead, Hertfordshire, UK) according to the manufacturer's instructions. Analysis was performed on a multiple analyte profiler (Luminex 100; MiraiBio Hitachi Genetic Systems, Alameda, CA, USA) with BioPlex software (Bio-Rad Laboratories, Hercules, CA, USA). The limits of accurate detection (read from the nonlinear portion of the standard curves) for each of the cytokines (in pg/mL) were as follows: 6.4–102.2 (IL-10), 34.7–555.9 (IP-10), 5.1–20.5 (MIP-1α) and 25.3–404.8 (TNF-α), with the lower values representing the lower limits of detection.

### Migration assay

Cells (2 × 10^5^; both unstimulated and cytokine-stimulated) were seeded into the upper wells of a 5-μm pore (Transwell; Fisher, Scientific UK, Loughborough, UK) filter. Either C-C motif ligand (CCL)19 (200 ng/mL) or bovine serum albumin (BSA, used as vehicle control), were added to 600 μL of RPMI medium in the lower well. Cultures were established in duplicate, and were incubated in a humidified atmosphere of 5% CO_2_ in air for 3 h at 37 °C. The number of migrated cells was counted using an automated cell counter (Casy Counter, Roche Applied Science, Burgess Hill, West Sussex, UK).

### Statistical analyses

Data were tested for normality and transformed via ranking, if required, using GraphPad Prism software (version 4.03; San Diego, USA). Released cytokine and chemokine data and surface marker expression data were analysed using two-way ANOVA followed by Bonferroni tests as *post hoc* tests. *P* < 0.05 was considered significant. All data are expressed either as individuals or as the mean value ± SEM, and are for 3–8 replicates as stated.

## Results

### Dendritic cell phenotype

**Figure 1:**
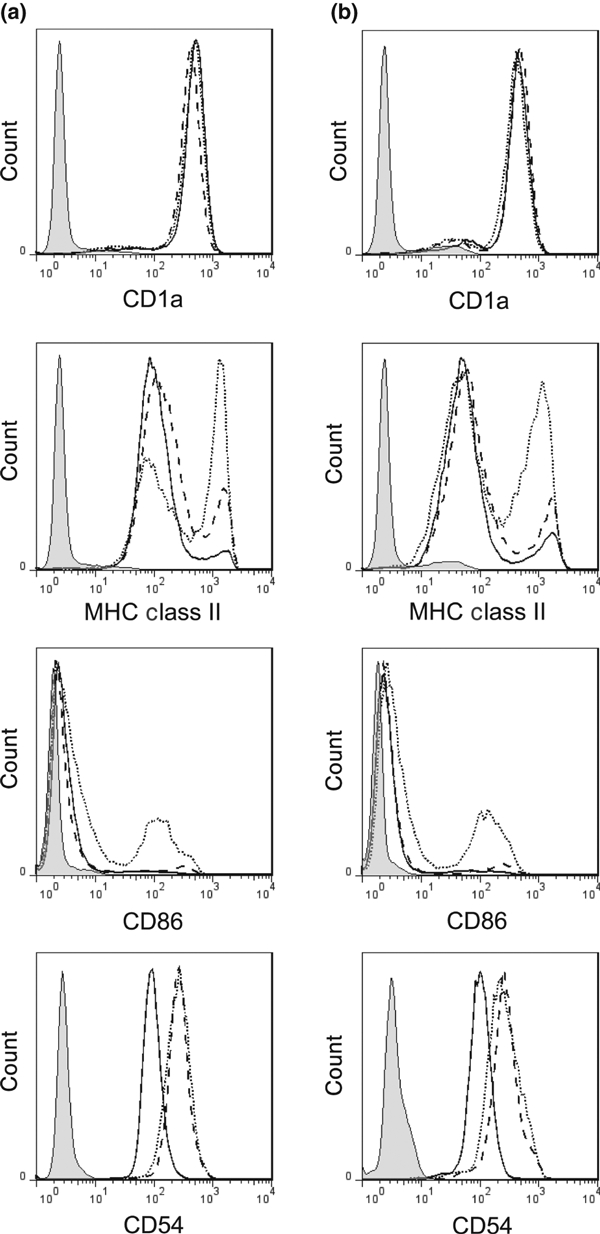
Representative histograms demonstrating mean fluorescence intensity of CD1a, CD86, CD54 and major histocompatibility complex class II by monocyte-derived Langerhans cells derived from both (a) healthy controls and (b) patients with early-onset psoriasis. Each histogram displays data for unstimulated (solid line) and cytokine-stimulated (dashed line, tumour necrosis factor-α; dotted line, interleukin-1β; both 100 ng/mL) cells, in addition to an isotype control (shaded histogram). MHC, major histocompatibility complex.

Following culture with GM-CSF, IL-4 and TGF-β, CD14+ monocytes derived from both healthy controls and patients with psoriasis acquired a DC-like phenotype, with CD14 expression decreasing to negligible levels in both groups. Concurrently, the percentage of CD1a+ cells at day 6 were 89.3 ± 3.8 and 88.9 ± 2.6%, in controls and patients, respectively, demonstrating that there was no impairment in the ability of monocytes obtained from patients with early-onset psoriasis to differentiate into DC-like cells (Fig. 1).

### Dendritic cell maturation following cytokine stimulation

**Figure 2:**
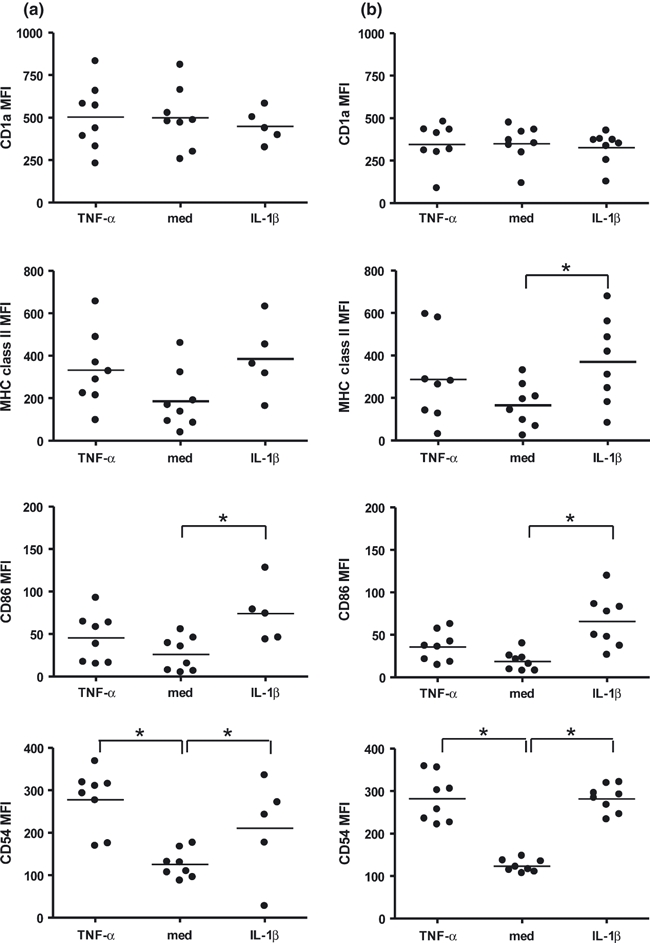
CD1a, CD86, CD54 and major histocompatibility complex class II expression by unstimulated (med) and cytokine-stimulated [interleukin (IL)-1β and tumour necrosis factor (TNF)-α; both 100 ng/mL] monocyte-derived Langerhans cells derived from both (a) healthy controls and (b) patients with early-onset psoriasis. Each individual is represented by a single point on the graph, with the mean displayed as a horizontal bar. *P < 0.05.

Baseline expression of surface markers as measured by flow cytometry was equivalent between mLCs derived from patients with psoriasis and from healthy controls (Figs 1,2). mLCs derived from patients with psoriasis were also able to respond to stimulation to the same extent as those derived from healthy volunteers, demonstrating that there was no impairment in their maturation ability (Figs 1, 2). For example, stimulation with IL-1β upregulated CD86 expression by approximately threefold in both the mLCs derived from patients and those derived from controls. CD54 expression was upregulated by both TNF-α and IL-1β to the same extent (approximately twofold) in both groups (*P* < 0.05; Fig. 2). Expression of MHC class II in both unstimulated and stimulated cells was equivalent between groups, and although there was a slight increase in this expression following stimulation, this was only significant for the patients following IL-1β stimulation (Fig. 2). There was a trend for CD1a intensity to be decreased in the mLCs derived from patients compared with those derived from healthy controls, but this was not significant (*P* > 0.05; Fig. 2).

### Secreted cytokine and chemokine analysis

**Figure 3:**
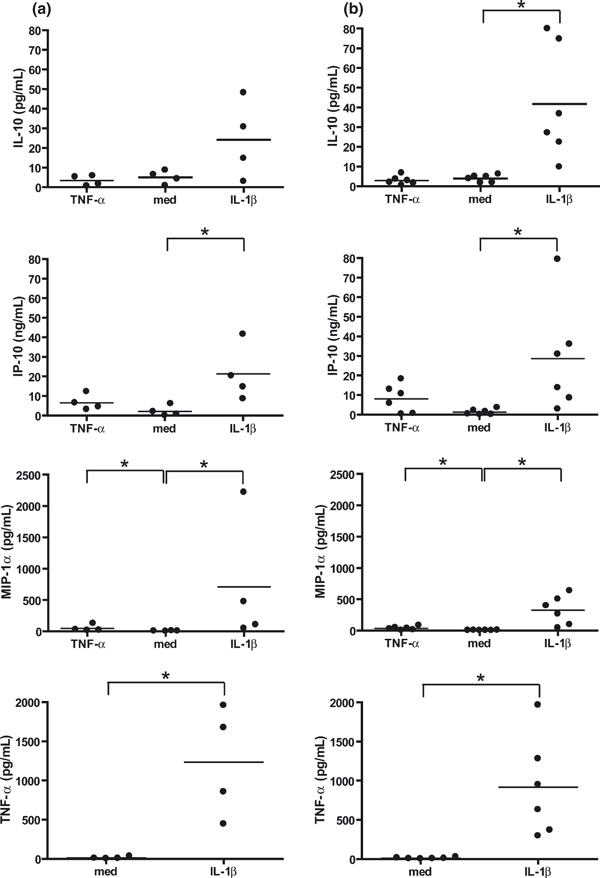
Quantification of secreted cytokines and chemokines from unstimulated (med) and cytokine-stimulated [interleukin (IL)-1β and tumour necrosis factor (TNF)-α; both 100 ng/mL] monocyte-derived Langerhans cells derived from both healthy controls (a) and patients with early-onset psoriasis (b). Each individual is represented by a single point on the graph, with the mean displayed as a horizontal bar. *P < 0.05. IP-10, interferon γ-induced protein-10; MIP-1α, macrophage inflammatory protein-1α.

These preliminary results demonstrate that there were no differences in baseline concentrations of any of the cytokines and chemokines analysed in the supernatants of mLCs derived from patients with psoriasis compared with those from healthy controls (Fig. 3). Stimulation with IL-1β increased release of all of the cytokines and chemokines analysed (IL-10, IP-10, MIP-1α and TNF-α) to similar extents in both groups (*P* < 0.05), with the exception of IL-10 in the mLCs derived from healthy controls, although this lack of statistical significance was thought to be due to the low number of replicates in this group rather than to a real physiological effect. In addition, stimulation with TNF-α increased MIP-1α to the same extent in both groups (*P* < 0.05). Thus, there were no differences in concentrations of any of the analytes following stimulation between mLCs derived from both patients with psoriasis and healthy controls (*P* > 0.05).

### Transwell migration

**Figure 4:**
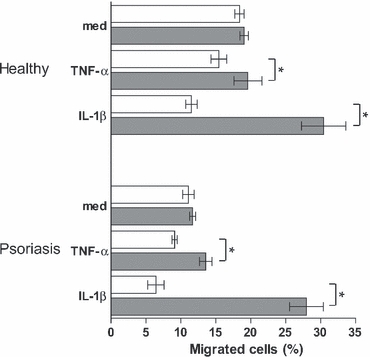
Percentage of unstimulated (med) and cytokine-stimulated [interleukin (IL)-1β and tumour necrosis factor (TNF)-α; both 100 ng/mL] cells that migrated to media supplemented with C-C motif ligand (CCL)19; (200 ng/mL; shaded bars) and bovine serum albumin (BSA; open bars), in both healthy controls and patients with early-onset psoriasis. Data are expressed as mean ± SEM, and are for *n* = 3 or 4 per group. *P < 0.05 for CCL19 compared with BSA (*P* < 0.05).

Stimulation with both TNF-α and IL-1β induced migration to CCL19 in both healthy individuals and patients with psoriasis (*P* < 0.05; Fig. 4), with the effect being more pronounced in IL-1β-stimulated samples. The baseline migration of mLCs to the vehicle control was lower in the mLCs derived from the patients compared with the healthy controls, in both unstimulated and stimulated (TNF-α and IL-1β) cells (*P* < 0.05). The fold increases in migration to CCL19 compared with BSA were equivalent between the mLCs derived from patients compared with those obtained from healthy controls, following either TNF-α or IL-1β stimulation (*P* > 0.05). The level of migration in response to CCL19 of TNF-α-stimulated cells was decreased in cells derived from patients compared with those derived from healthy controls (13.5 ± 0.9% and 19.6 ± 2.0 in mLCs derived from patients and controls, respectively; *P* < 0.05). However, this same effect was not observed in the cells stimulated with IL-1β, in which levels of migration were equivalent between the two groups (27.9 ± 2.4% and 30.4 ± 3.2 in mLCs derived from patients and controls, respectively; *P* > 0.05).

## Discussion

It is known that in the uninvolved skin of patients with early-onset psoriasis, there is a complete inhibition of LC migration in response to relevant cytokine stimulation.^10^ We therefore wanted to investigate whether this impairment of LC function in the skin is paralleled by changes in the phenotype and function of other DC members. To address this, we examined the characteristics of LC-like cells derived from peripheral blood monocytes isolated from patients with early-onset psoriasis, and compared them with mLCs derived from healthy controls matched for age and gender. The results demonstrated that there were no clear phenotypic or functional differences in mLCs derived from patients with early-onset psoriasis compared with healthy controls. This suggests that the impairment in LC function observed *in vivo* is not indicative of a generalized defect in DC biology, but rather is either cell-type specific or due to abnormalities in the skin microenvironment.

A number of diseases, including Alzheimer's disease,^18^ systemic lupus erythematosus^19^,^20^ and rheumatoid arthritis,^21^ have been associated with changes in the phenotypic and functional characteristics of monocyte-derived DCs (i.e. CD14+ monocytes cultured in the presence of GM-CSF and IL-4 alone). Considering the systemic nature of psoriasis, it is reasonable to question the involvement of circulating DCs in the pathogenesis of this skin disease. In addition to no differences being found in mLCs, we also did not observe any obvious systemic inflammation, in that there were equivalent numbers of both PBMCs and monocytes in the blood drawn from both groups. However, it is possible that any potential increases in monocyte numbers in the patients with psoriasis may have been masked by their enhanced migration to inflamed skin.^22^

There was a tendency for a decrease in CD1a expression in the mLCs derived from the patients compared with healthy controls, although this was not significant. A reduction in CD1a expression is indicative of DC maturation, and DCs isolated from plaques of psoriasis have previously been demonstrated to possess a mature phenotype, in terms of a loss of CD1a.^23^ CD1a is decreased in mLCs derived from healthy controls that have been incubated with the supernatants of cultured keratinocytes isolated from plaques of psoriasis, compared with keratinocytes isolated from healthy skin.^24^ This indicates that an alteration in the skin microenvironment in patients with psoriasis is able to influence LC biology.

Interestingly, there was a general suppression in migratory capacity in the mLCs derived from patients with psoriasis compared with those from healthy controls, with the exception of the IL-1β-stimulated cells, in which migratory capacity was equivalent between the two groups. It is unclear as to why background migration decreased following stimulation with TNF-α and IL-1β in both groups. One possible explanation is that a stimulation-induced reorganization of the cytoskeleton was sufficient to inhibit this nonspecific migration. These effects on background migration are of interest, and warrant further investigation. The unstimulated, immature mLCs did not migrate to CCL19, as expected^25^ because CCL19 is a ligand for CCR7, which is only upregulated upon activation. Although CCL19 is generally associated with DC migration to lymph nodes, Robbiani *et al*. reported that antagonism of CCL19 prevented migration of LCs from the epidermis following exposure to FITC.^26^

There was an equivalent release of cytokines and chemokines between the two groups, demonstrating that there is no impairment in the ability of mLCs derived from patients with early-onset psoriasis to respond to cytokine stimulation. Chemokines and chemokine ligands have been suggested to play a role in DC and T-cell positioning within the skin. Zhou *et al*. found that chemokines such as CCL19, chemokine (C-X-C motif) ligand (CXCL)12 and monocyte chemotactic protein-1 are upregulated in plaques of psoriasis compared with skin from healthy individuals, although it is unclear from the study as to whether there was any alteration in the uninvolved skin.^27^ Quantification of chemokines and ligands within the uninvolved skin would thus be of interest with respect to the retention of LCs within uninvolved skin.

## Conclusions

The results of this investigation demonstrate that there are neither phenotypic nor functional differences in mLCs derived from patients with early-onset psoriasis compared with those derived from healthy controls. Thus, the impaired epidermal LC function observed *in vivo* does not appear to represent a genetic defect in DCs, but rather suggests an aberration in the skin microenvironment.

**What is already known about this topic?**

- Epidermal LC function is impaired in the uninvolved skin of patients with early-onset psoriasis.
- Several diseases have demonstrated associations with changes in the phenotype and function of monocyte-derived dendritic cells.

**What does this study add?**

- The impaired LC function observed in patients with psoriasis is not reflective of a systemic defect in monocyte-derived DCs and precursors of LCs.
- The implication is that in the absence of a systemic functional impairment of DCs, the defect observed in LC mobilization in psoriasis is specifically associated with the LC/epidermal axis.
